# Does kisspeptin exert a local modulatory effect on bovine ovarian steroidogenesis?

**DOI:** 10.1530/RAF-22-0088

**Published:** 2023-02-23

**Authors:** Dareen Mattar, Warakorn Cheewasopit, Moafaq Samir, Phil G Knight

**Affiliations:** 1 Department of Physiology, College of Medicine, Umm Al-Qura University, Makkah, Saudi Arabia; 2 Department of Biology, Ramkhamhaeng University, Bankapi, Bangkok, Thailand; 3 The Ministry of Higher Education and Scientific Research, Baghdad, Iraq; 4 School of Biological Sciences, University of Reading, Whiteknights, Reading, UK

**Keywords:** kisspeptin, ovary, granulosa cell, theca cell, steroidogenesis

## Abstract

Kisspeptin, a hypothalamic neuropeptide encoded by the *KISS1* gene, has a pivotal role in promoting gonadotrophin-releasing hormone secretion in mammals. Kisspeptin and its receptor (*KISS1R*) are also expressed in certain peripheral tissues including gonads, suggesting intra-gonadal roles. Such actions at the level of the bovine ovary have not been explored previously. The current aims were to determine whether *KISS1* and *KISS1R* are expressed in the bovine ovary and whether kisspeptin or a kisspeptin antagonist can modulate ovarian steroid production by cultured ovarian cells. Granulosa cells (GC) and theca interna cells (TC) were collected from antral follicles (3–18 mm) categorized into five class sizes. Early, mid and regressing corpora lutea (CL) were also collected for RT-qPCR analysis of *KISS1* and *KISS1R* expression. Bovine TC and GC cultured under both non-luteinizing (serum-free) and luteinizing (serum-supplemented) conditions were treated for 4 days with kisspeptin-10 (10^–^^10^–10^–^^6^M) or kisspeptin antagonist (kp234; 10^–10^–10^–6^M), alone and in combination with either follicle-stimulating hormone (GC), luteinizing hormone (TC) or forskolin (luteinized GC/TC). Steroid secretion (GC: oestradiol, progesterone; TC: androstenedione, progesterone; luteinized GC/TC: progesterone) was measured by ELISA and viable cell number determined by neutral red uptake assay. *KISS1* and *KISS1R* transcripts were detected in TC, GC and CL with significant differences between follicle categories and CL stages. However, neither kisspeptin-10 nor kisspeptin antagonist affected steroid secretion or viable cell number in any of the four ovarian cell culture models. As such, the hypothesis that kisspeptin has a direct intraovarian role to modulate follicular or luteal steroidogenesis, or cell proliferation/survival, is not supported.

**Lay summary:**

Kisspeptin-producing nerve cells (neurones) in the hypothalamus play a crucial role in controlling the reproductive system. Kisspeptin activates receptors on gonadotrophin-releasing hormone neurones which, in turn, stimulate the pituitary gland to release gonadotrophins. Gonadotrophins act on the gonads (ovaries or testes) to regulate their function (e.g. ovarian follicle development and steroid production). Evidence has emerged in several species that kisspeptin and its receptor are also present in certain peripheral tissues, including the ovaries, suggesting ‘local’ actions. So far, few studies have investigated this. Here, we first show that both kisspeptin and its receptor are expressed by key ovarian cell types of cattle. However, we found that treating cultured bovine theca and granulosa cells with kisspeptin or kisspeptin antagonist did not modify steroid secretion. As such, the hypothesis that kisspeptin has a direct intraovarian role to modulate ovarian steroid production is not supported.

## Introduction

Kisspeptin, encoded by the* KISS1* gene, is a potent endogenous secretagogue of GnRH, acting via its cognate receptor, KISS1R (aka GPR54), expressed by many hypothalamic GnRH neurones (Roseweir & Millar 2009, Smith *et al.* 2006). Kisspeptin–KISS1R signalling promotes pulsatile gonadotropin secretion that, in turn, drives follicular development, steroidogenesis and ovulation in the female and steroidogenesis and spermatogenesis in the male (Ohkura *et al.* 2009, Roseweir & Millar 2009). Central or peripheral administration of kisspeptin stimulates GnRH secretion in mouse (Han *et al.* 2005), rat (Irwig *et al.* 2004, Castellano *et al.* 2005), sheep (Messager *et al.* 2005), monkey (Shahab *et al.* 2005, Plant *et al.* 2006) and human (Dhillo *et al.* 2005), and it seems highly probable that similar mechanisms operate in the bovine (Naniwa *et al.* 2013). Hypothalamic kisspeptin-expressing neurones are located in the anteroventral periventricular nucleus, periventricular nucleus, anterodorsal preoptic nucleus and arcuate nucleus (Han *et al.* 2005, Smith *et al.* 2005, 2006). The *KISS1* gene encodes a precursor peptide that is further processed to generate biologically active forms of kisspeptin of different size (10, 13, 14 and 54 amino acids), all of which share the minimal C-terminal sequence required for receptor activation (Oakley *et al.* 2009).

Although an abundance of evidence indicates that KISS1–KISS1R signalling occurs primarily at the hypothalamic level to regulate GnRH output, there is mounting evidence that kisspeptin and/or its receptor are expressed at other levels of the reproductive system, including the gonads (Gaytan *et al.* 2009, Naniwa *et al.* 2013, Gaytan *et al.* 2014, Merhi *et al.* 2016, Hu *et al.* 2017, Cao *et al.* 2019). Ovarian expression of KISS1 and KISS1R mRNA and/or protein has been reported in various species, including human, marmoset monkey, rat, cat, dog and pig (Castellano *et al.* 2006, Gaytan *et al.* 2009, Zhou *et al.* 2014, Cielesh *et al.* 2017, Basini *et al.* 2018, Tanyapanyachon *et al.* 2018). Expression of *Kiss1* mRNA by cultured rat granulosa cells (GCs) was upregulated by human chorionic gonadotrophin (hCG) (Laoharatchatathanin *et al.* 2015). To our knowledge, information is lacking on the extent to which kisspeptin and its receptor are expressed in the ovary and other endocrine tissues of cattle.

In rats, kisspeptin and kisspeptin receptor expression was detected in ovarian tissues, with expression levels evidently fluctuating in a cyclic-dependent manner under the control of pituitary luteinizing hormone (LH) (Castellano *et al.* 2006). In good agreement, studies on the Siberian hamster documented that kisspeptin immunoreactivity increased through the ovulatory transition including pro-estrus and estrus (Shahed & Young 2009). Exposure of rats to a high-fat diet led to a reduced expression of kisspeptin in the theca interna cell (TC) layer of antral follicles during estrus and proestrus (Zhou *et al.* 2014). Expression levels of *KISS* and *KISS1R* mRNA in granulosa–lutein cells were higher in women with polycystic ovarian syndrome (PCOS) compared with non-PCOS women (Hu *et al.* 2019). With regard to steroidogenesis, it was reported that progesterone (P4) production by rat luteal cells was stimulated by kisspeptin (Peng *et al.* 2013), while hCG-induced P4 production by rat GC was attenuated by a kisspeptin antagonist, kisspeptin 234 (Laoharatchatathanin *et al.* 2015). Kisspeptin was also shown to increase P4 production by porcine GC (Basini *et al.* 2018) and bovine GC (Guo *et al.* 2022). The above reports suggest a potential role of locally produced kisspeptins in the control of ovarian function. However, the physiological relevance of an intraovarian kisspeptin–kisspeptin receptor system remains underexplored to date, and more functional studies are needed to evaluate potential effects on different aspects of ovarian function, including TC steroidogenesis.

The aims of this study were first to investigate whether mRNAs encoding *KISS1* and its receptor (*KISS1R*) are expressed in the bovine ovary and other endocrine tissues (pituitary, adrenal and testis) and secondly, to investigate the effects of kisspeptin and a kisspeptin antagonist (alone and in combination) on ovarian steroidogenesis *in vitro*. For the latter, we used four different primary ovarian cell culture systems (bovine TCs and GCs under non-luteinized and luteinized conditions) designed to represent both preovulatory (i.e. follicular) and postovulatory (i.e. luteal) stages.

## Materials and methods

Unless otherwise state, all materials were purchased from Sigma UK Ltd. or Life Technologies Ltd.

### Bovine tissue collection and ovarian cell culture

Bovine ovaries from randomly cycling cattle and other endocrine tissues (pituitary glands, testes and adrenal glands) were obtained from an abattoir. Ovaries were dissected to obtain antral follicles ranging in diameter from 3 mm to 18 mm, and these were further processed to isolate the GC layer, TC layer, and follicular fluid, as described previously (Glister *et al.* 2010). Briefly, follicles were sorted into five different size classes: 3–4 mm (*n* = 8), 5–6 mm (*n* = 8), 7–8 mm (*n* = 9), 9–10 mm (*n* = 6) and 11–18 mm (*n* = 12). Each follicle was hemisected, and GC and TC layers were recovered for RNA extraction, while follicular fluid was recovered for steroid hormone analysis. Large follicles (11–18 mm) were reclassified according to their oestrogen to progesterone ratio (E:P ratio) in follicular fluid as either large oestrogen-active (LEA; E2:P4 ratio >1) or large oestrogen-inactive (E2:P4 ratio <1) follicles. Corpora lutea (CL) at growing (*n* = 4), mid-luteal (*n* = 5) and regressing (*n* = 4) stages were also harvested. All tissue samples were homogenized in Trizol reagent for total RNA extraction, as described previously (Glister *et al.* 2010).

Follicles (4–8 mm diameter) were also retrieved for isolation of GC and TC to be used for primary cell culture experiments, as described in detail elsewhere (Glister *et al.* 2001, 2005). GC and TC were seeded into 96-well plates (Nunclon, Life technologies Ltd.) at a density of 75,000 cells/250μL/well for serum-free culture (non-luteinized cells) or 10,000 cells/250 μL/well for serum-supplemented culture (luteinized cells). Cells were cultured for 6 days at 38.5°C with saturating humidity in 5% CO_2_ in air. The culture medium consisted of McCoy’s 5A medium (Sigma), supplemented with antibiotic/antimycotic solution (1% v/v; Sigma), apo-transferrin (5 μg/mL; Sigma), sodium selenite (5 ng/mL; Sigma), bovine insulin (10 ng/mL; Sigma), HEPES (20 mM; Sigma) and bovine serum albumin (0.1% w/v; Sigma). Medium used for serum-free GC culture was also supplemented with 10^–7^ M androstenedione as aromatase substrate. For GC and TC cultured under conditions that promote luteinization, 2% fetal calf serum (FCS) was also included as a supplement. In all four culture models, media were changed after 48 h and replaced with fresh media containing treatments as specified below. This was repeated after a further 48-h incubation period. Media were retained after the final 48-h period (i.e. 96–144 h) for subsequent analysis of steroid hormone secretion. Viable cell number at the end of culture was determined by neutral red uptake, as described elsewhere (Glister *et al.* 2001).

It should be noted that culturing TC and GC using defined serum-free medium preserves a non-luteinized phenotype reflected by LH-induced androstenedione (A4) secretion by TC and follicle-stimulating hormone (FSH)-induced oestradiol (E2) secretion by GC. Henceforth, these cells will be referred to as non-luteinized TC (NLTC) and non-luteinized GC (NLGC). In contrast, culturing TCs and GCs under serum-supplemented conditions promotes spontaneous luteinization, as indicated by reduced A4/E2 secretion and greatly increased secretion of P4 (Glister *et al.* 2001, 2005, Kayani *et al.* 2009). Henceforth, these cells will be referred to as LTC and LGC.

### Cell culture treatments and experimental design

Kisspeptin-10 (rat) and kisspeptin antagonist (p234) were purchased from Tocris Bioscience (Abingdon, Oxon, UK). Kisspeptin-10 corresponds to the C-terminal region of the translated kisspeptin 54 peptide (residues 112–121), and its sequence is identical in rat and bovine. Kisspeptin-10 and p234 (antagonist) were dissolved in water and 20% (w/v) acetonitrile in water, respectively, to give a stock concentration of 10^–3^ M. These were further diluted in sterile medium to give final concentrations of 10^–6^, 10^–7^, 10^–8^, 10^–9^ and 10^–10^ M. In each culture model, the effects of kisspeptin-10 and kisspeptin antagonist on steroid production and viable cell number were evaluated under both basal and gonadotrophin or forskolin (FSK)-stimulated conditions as explained below. Highly purified ovine FSH (oFSH 19SIAPP) and LH (oLH-S-16) were provided by the NHPP (Torrance, CA, USA). In NLTC cultures, LH was used at a final concentration of 150 pg/mL, shown previously to elicit maximal A4 secretion (Glister *et al.* 2005); A4 and P4 secretion were evaluated. In NLGC cultures, FSH was used at a final concentration (0.3 ng/mL) shown previously to elicit optimal E2 secretion (Glister *et al.* 2001, 2004); E2 and P4 secretion were evaluated. As LGC and LTC are largely unresponsive to gonadotrophin stimulation in this serum-supplemented culture model (Kayani *et al.* 2009), the adenylate cyclase activator, forskolin (FSK; 10 μM), was used as an alternative secretagogue; only P4 secretion was evaluated for these cells since A4 and E2 secretion are both extremely low.

### Gene expression analysis by RT-qPCR

For each sample, cDNA was synthesized from 1 μg total RNA using the AB High Capacity cDNA synthesis kit with random hexamer primers (Thermo Fisher Scientific Ltd) and used for SYBR Green-based real-time PCRs performed, as described previously (Glister *et al.* 2010), using the specific primer pairs shown in Table 1. Briefly, real-time PCRs (14 μL) included 5 μL of diluted cDNA sample, 2 μL of forward and reverse primer mix and 7 μL of QuantiTect SYBR Green 2× Master Mix (Qiagen) and were run for 40 cycles on an AB StepOne plus real-time PCR instrument (Thermo Fisher Scientific). The ∆∆C_t_ method (Livak & Schmittgen 2001) was used to evaluate relative transcript abundance using β-actin (*ACTB*) as the housekeeping control. Resultant ∆C_t_ values for individual replicates within each tissue group were then normalized to the average ∆C_t_ value of these different tissues to give ∆∆C_t_ values. Finally, ∆∆C_t_ values were converted to fold difference for graphical presentation using the formula 2^(-∆∆Ct)^.

**Table 1:** List of primers used for quantitative RT-PCR.

Target	Accession number	Forward primer 5′–3′	Reverse primer 5′–3′	Amplicon size (bp)
***KISS1***	AB_466319.1	AAGGCAAGGGCACTTCCAAGACC	TTTCCAGTGTCTCCCTGAAGGCG	110
***KISS1R***	XM_003582417.2	TGTTGCTCGGGTGAACAGTGG	AGCCACTGCGCGTTTATACCCC	112
***ACTB***	NM_173979.3	ATCACCATCGGCAATGAGCGGTTC	CGGATGTCGACGTCACACTTCATGA	128

### Hormone immunoassays

A4, E2 and P4 concentrations in media samples were determined by direct competitive ELISA as reported previously (Glister *et al.* 2005, 2013, 2014). Within- and between-plate coefficient of variations were <9 and <12%, respectively, for each assay.

### Statistical analysis

Comparison of relative gene expression amongst different tissues and follicular stages were made by one- or two-way analysis of variance (ANOVA) using ∆C_t_ values before conversion to fold differences. To reduce the heterogeneity of variance, hormone secretion data were log-transformed prior to statistical analysis using one or two-way (ANOVA. Individual pairwise comparisons between different groups were made by Fisher’s protected least significant difference test. Cell culture results are presented as arithmetic means (+s.e.m.) based on ≥3 independent batches of cells.

## Results

### The expression of KISS1 and KISS1R in different bovine endocrine tissues

**Figure 1:**
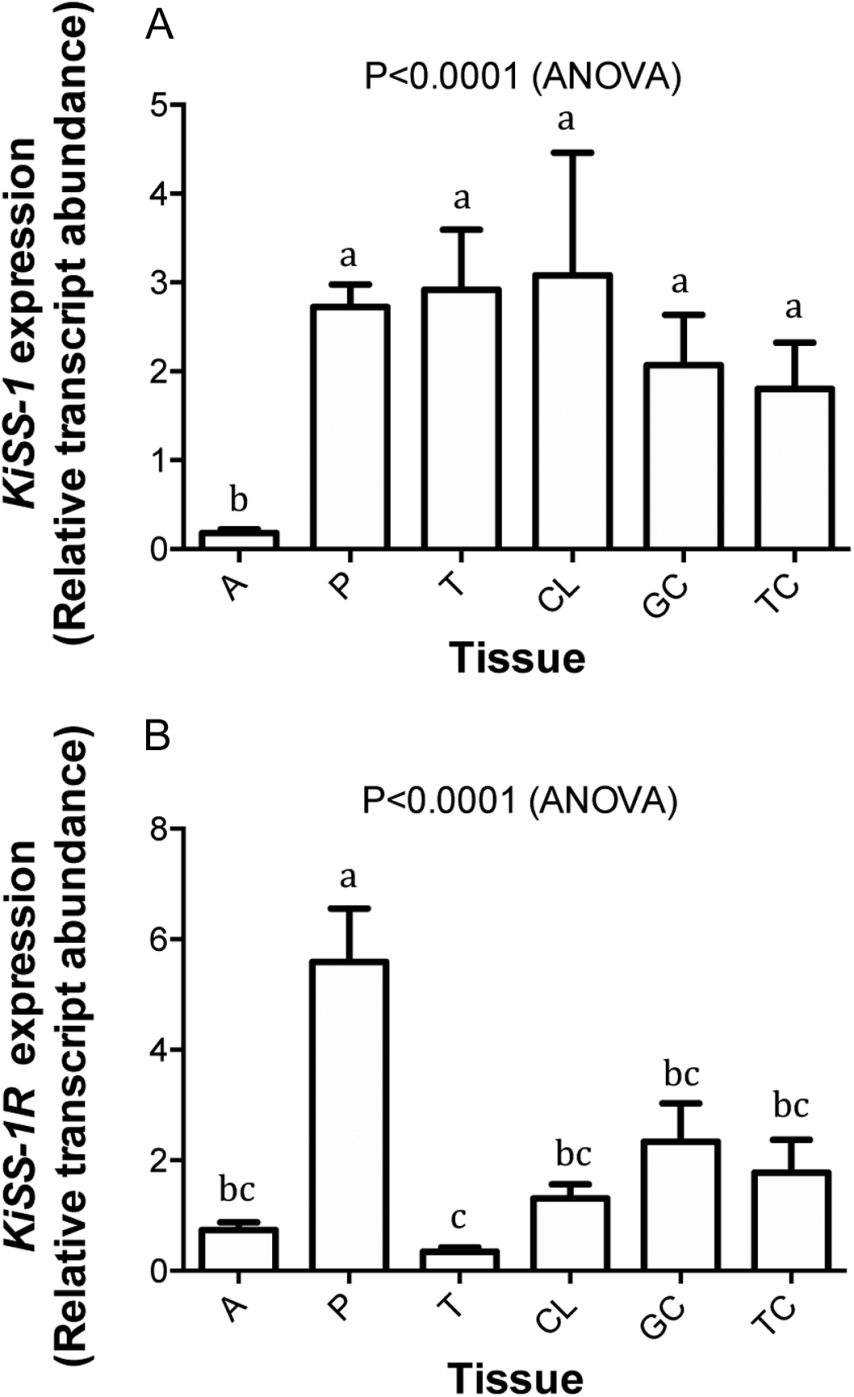
Comparison of the relative abundance of mRNA transcripts for (A) *KISS1* and (B) *KISS1R* (*GPR54*) in different bovine endocrine tissues including adrenal gland (A, *n* = 6), pituitary gland (P, *n* = 6), testis (T, *n* = 6), corpus luteum (CL, *n* = 13), granulosa cells (GC, *n* = 38), and theca cells (TC, *n* = 43) and bars indicate s.e.m.; means without a common letter are significantly different (*P* < 0.05).

The expression of *KISS1* in different bovine endocrine tissues including pituitary, adrenal, testis and ovary (TC, GC and CL) varied significantly (*P* < 0.0001), with adrenal tissue having the lowest level (Fig. 1A). Also, the expression of *KISS1R* varied significantly (*P* < 0.0001), being highest in pituitary and lowest in testis (Fig. 1B).

**Figure 2:**
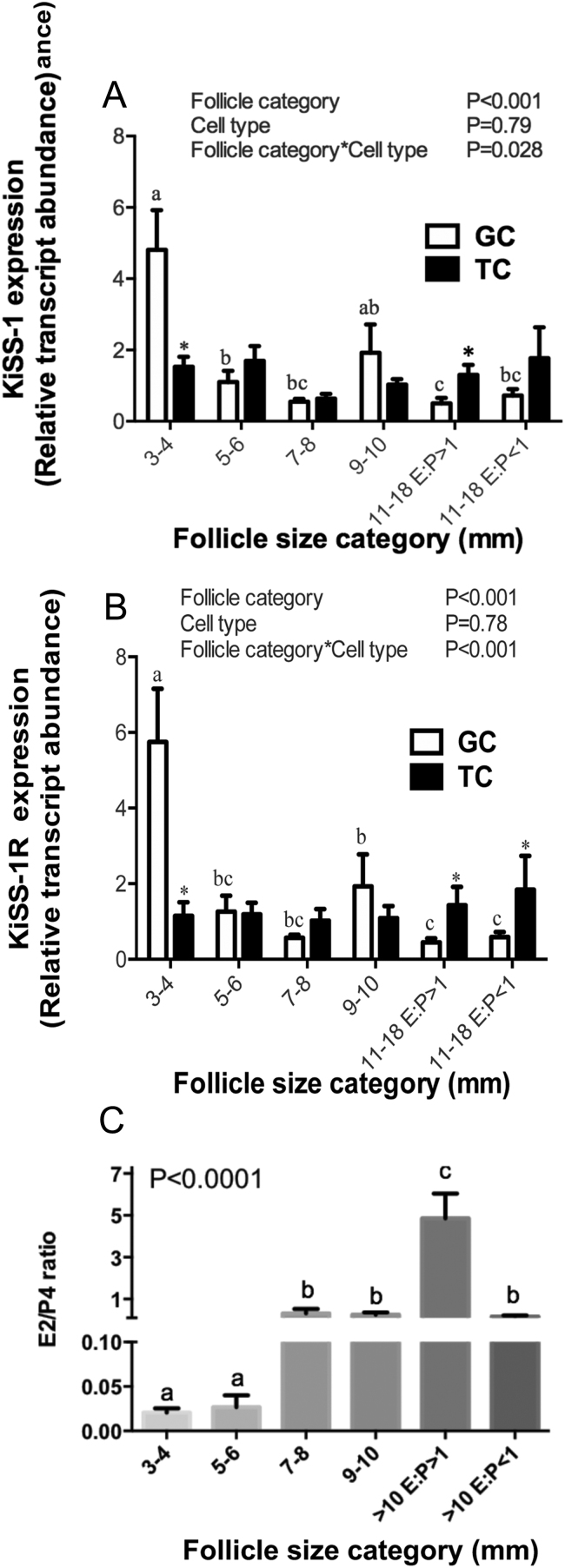
Comparison of the relative abundance of mRNA transcript for (A) *KISS1* and (B) *KISS1R* in GC and TC from ovarian follicles classified according to size. Follicles in the largest size category were subdivided on the basis of their intrafollicular oestrogen to progesterone ratio (E2:P4 >1 or E2:P4 <1) shown in panel (C). Values are means and bars indicate s.e.m.; summarized ANOVA results are shown. Open bars (GC) without a common letter are significantly different (*P* < 0.05); Expression levels in TC did not differ between different size categories (*P* > 0.05). *P < 0.05 TC group vs corresponding GC group.

Expression of *KISS1* in ovarian follicles varied according to follicle category, with maximum expression observed in GC of the smallest size class analysed (Fig. 2A). Likewise, the expression of *KISS1R* was also significantly affected by follicle size class and was highest in GC of the smallest follicles (Fig. 2B). Whilst *KISS1* and *KISS1R* expression levels in TC did not differ significantly amongst different follicle classes, both transcripts showed significant differences between TC and GC. In the smallest follicle class, both *KISS1* and *KISS1R* transcripts were much more abundant in GC than TC, whereas this pattern was reversed in the largest follicle class. Expression levels of both transcripts in large (11–18 mm) follicles did not differ between those classified as oestrogen active (E2:P4 ratio >1) and oestrogen inactive (E2:P4 ratio <1). Intrafollicular E2:P4 ratios for all follicle categories are shown in Fig. 2C.

**Figure 3:**
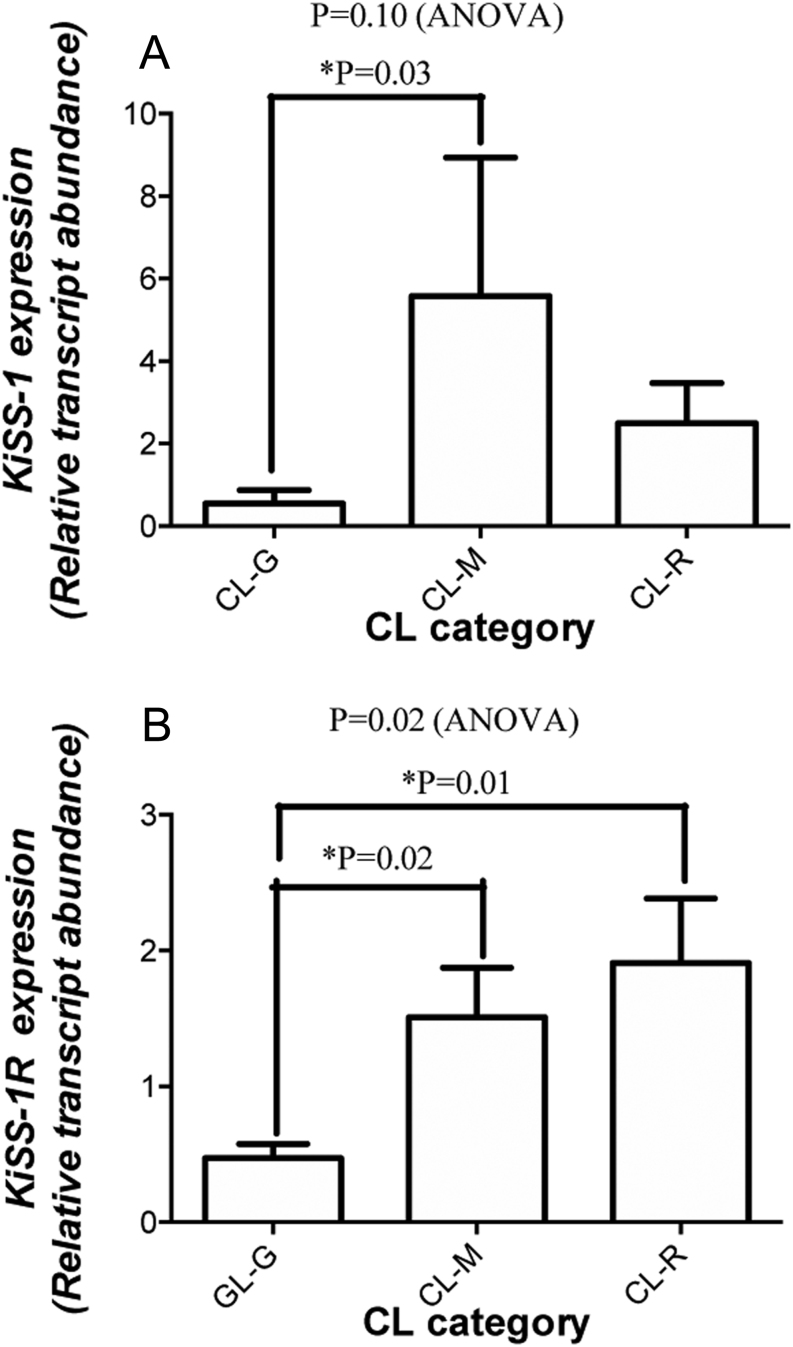
The expression of (A) *KISS1* and (B) *KISS1R* in CL tissue at growing (G, *n* = 4), mid-luteal (M, *n* = 5) and regressing (R, *n* = 4) stages. Values are means and bars indicate s.e.m.; results of pairwise comparisons are shown.

As shown in Fig. 3, the expression of both *KISS1* and *KISS1R* in CL tissue was significantly greater in the mid-luteal stage compared with the early (growing) luteal stage. While *KISS1* expression declined in regressing CL (n.s. vs early-stage CL), *KISS1R* expression remained high (*P* < 0.01 vs early-stage CL).

### Lack of effect of kisspeptin-10 and kisspeptin antagonist on basal and LH-induced A4 and P4 secretion by non-luteinized bovine TC

**Figure 4:**
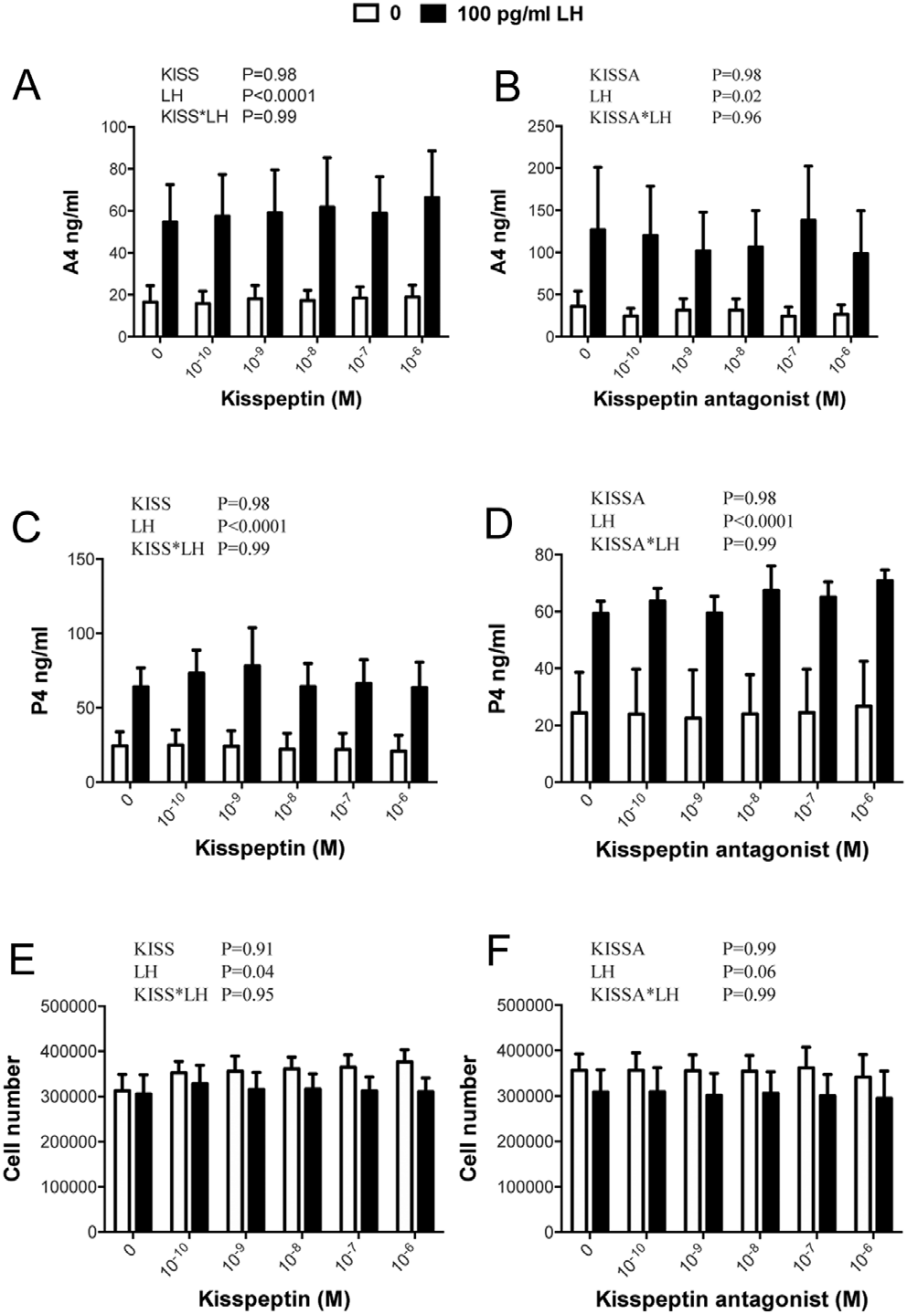
Lack of effect of kisspeptin-10 (left) and kisspeptin antagonist (right) on basal and LH-induced production of (A, B) A4 and (C, D) P4 by non-luteinized TC; lower panels (E, F) show viable cell number at the end of the culture. Values are means and bars indicate s.e.m. (*n* = 6 independent batches of cells); two-way ANOVA results are shown.

LH promoted a significant increase in secretion of both A4 and P4 by non-luteinized TC and a small though significant decrease in viable cell number at the end of culture. However, neither basal nor LH-stimulated production of A4 and P4 was affected by kisspeptin-10 or kisspeptin antagonist (Fig. 4). Likewise, there was no effect on viable cell number at the end of the culture period.

### Lack of effect of kisspeptin-10 and kisspeptin antagonist on basal and FSH-induced E2 and P4 secretion by non-luteinized bovine GC

**Figure 5:**
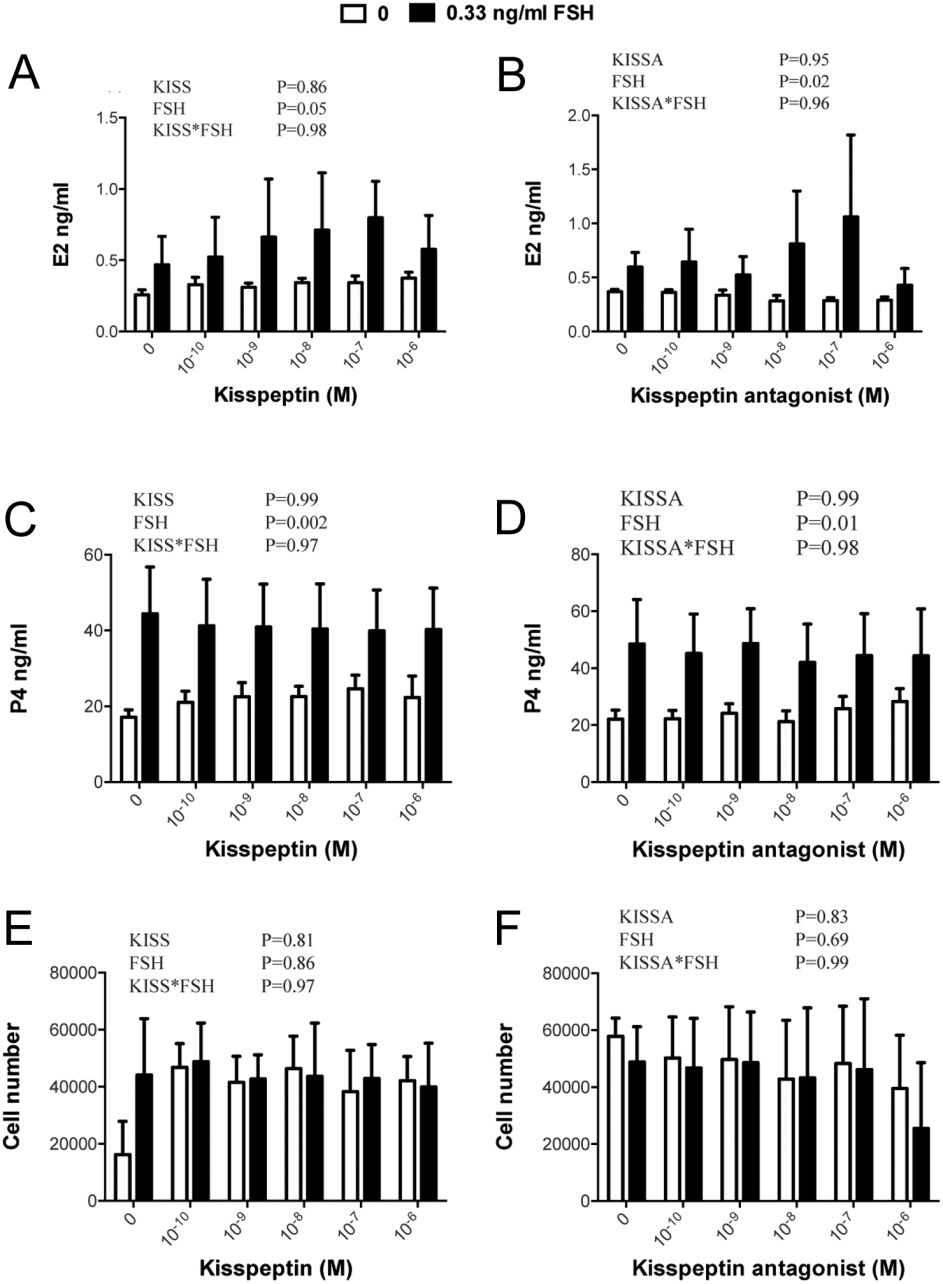
Lack of effect of kisspeptin-10 (left) and kisspeptin antagonist (right) on basal and FSH-dependent production of (A, B) E2 and (C, D) P4 by non-luteinized bovine GC; lower panels (E, F) show viable cell number at the end of the culture. Values are means, and bars indicate s.e.m. (*n* = 3 independent batches of cells); two-way ANOVA results are shown.

As shown in Fig. 5, basal and FSH-stimulated production of E2 and P4 by non-luteinized GC were not affected by either kissspeptin-10 or kisspeptin antagonist. Likewise, there was no effect on viable cell number at the end of the culture period.

### Kisspeptin-10 and kisspeptin antagonist do not affect basal or FSK-induced secretion of P4 by luteinized TC or GC

**Figure 6:**
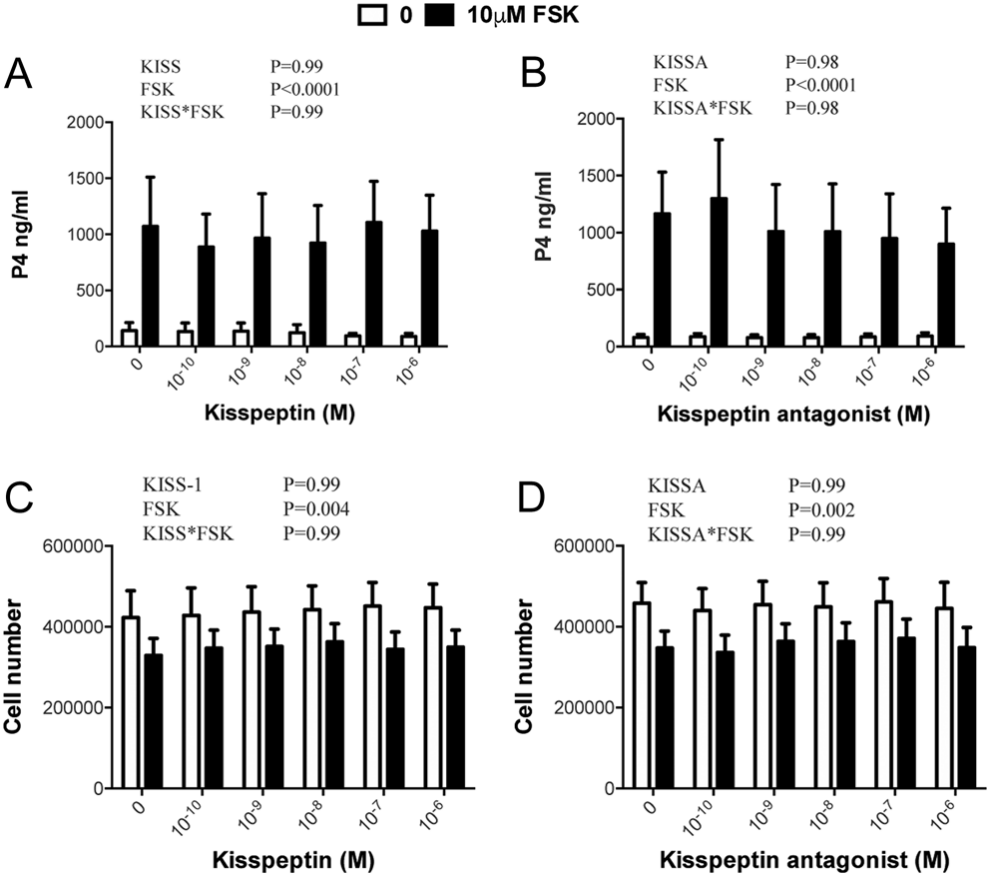
Lack of effect of kisspeptin-10 (A, B) and kisspeptin antagonist (C, D) on basal and FSK-stimulated production of P4 by luteinized bovine TC (A, C) and on viable cell number at the end of the culture (B, D). Values are means, and bars indicate s.e.m. (*n* = 5–6 independent batches of cells); two-way ANOVA results are shown.

**Figure 7:**
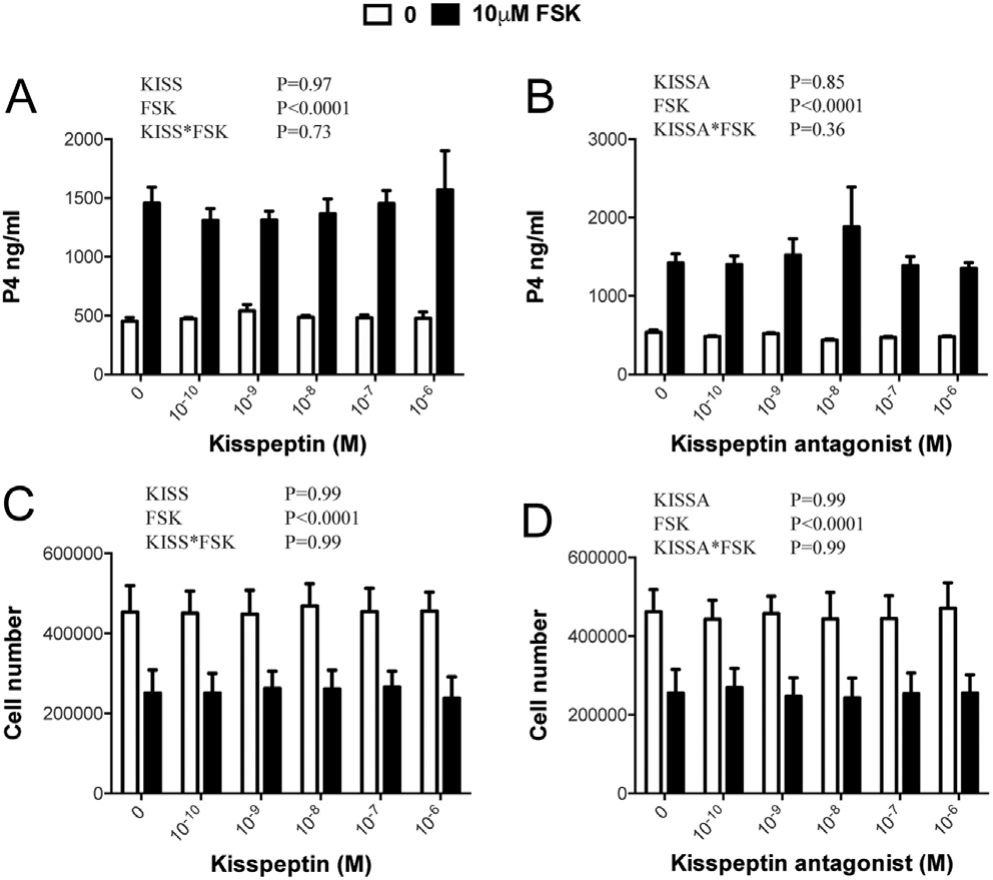
Lack of effect of kisspeptin-10 and kisspeptin antagonist on basal and FSK-stimulated production of P4 by luteinized bovine GC (A, C) and on viable cell number at the end of the culture (B, D). Values are means, and bars indicate s.e.m. (*n* = 3 independent batches of cells); two-way ANOVA results are shown.

As shown in Fig. 6, FSK promoted a substantial increase in P4 secretion by luteinized TC (*P* < 0.0001) and a small reduction in viable cell number (*P* = 0.004). However, kisspeptin-10 and kisspeptin antagonist did not modify basal or FSK-induced secretion of P4 or affect viable cell number. Similarly, kisspeptin-10 and kisspeptin antagonist did not modify basal or FSK-induced secretion of P4 by luteinized GC or change viable cell number at the end of the culture period (Fig. 7).

## Discussion

This study shows that *KISS1* and *KISS1R* (*GPR54*) mRNAs are expressed in different endocrine tissues of cattle, including pituitary, adrenal, testis and ovary, with ovarian expression identified in follicular TC and GC layers and in CL tissue. The adrenal gland showed a much lower level of the expression of *KISS1* than the other tissues examined. Expression of *KISS1R* varied significantly, being highest in pituitary and lowest in testis, with intermediate expression levels in the different ovarian compartments. More interestingly, this study revealed significant changes in the mRNA expression profiles of ovarian *KISS1* and *KISS1R* in TC and GC layers from follicles at different stages of development and in CL tissue at different stages of the luteal phase. In the rat ovary, the expression of kisspeptin and its receptor at the protein level was also reported to be stage dependent, with immunostaining detected in the theca layer of growing and preovulatory follicles from oestrus to early pro-oestrus and also in the GC layer of preovulatory follicles in late pro-oestrus (Castellano *et al.* 2006). This pattern appears to differ from that we observed in the cow ovary, where maximal expression of *KISS1* and *KISS1R* was observed in GC of small antral follicles (3–5 mm). Relative expression of both *KISS1* and *KISS1R* was much higher in GC than TC in these small bovine follicles, whereas this ratio was reversed in large follicles (11–18 mm diameter), regardless of their oestrogenic status. After ovulation in the rat, the expression of Kiss1 and Kiss1r was found in the theca–lutein cells of the corpus luteum and expression decreased as the corpus luteum regressed (Castellano *et al.* 2006, Roseweir & Millar 2009). The latter observation concurs with the reduced *KISS1* mRNA expression observed in regressing bovine CL in the present study.

Ovarian kisspeptin expression was first reported in rats (Terao *et al.* 2004, Castellano *et al.* 2006) with expression of kisspeptin and its receptor found in TCs, interstitial tissue and CL, but not in GCs, in contrast to the present findings in the bovine. However, there are some inconsistencies amongst studies, even with the same species, regarding cellular expression of kisspeptin and its receptor (Terao *et al.* 2004, Castellano *et al.* 2006, Shahed & Young 2009, Laoharatchatathanin *et al.* 2015, Garcia-Ortega *et al.* 2016, Mondal *et al.* 2016). For instance, reports of an apparent absence of Kiss1 and Kiss1r immunoreactivity in the GC of rat ovary (Castellano *et al.* 2006, Zhou *et al.* 2014) contrast with reports of high expression in other studies (Ricu *et al.* 2012, Peng *et al.* 2013, Laoharatchatathanin *et al.* 2015, Basini *et al.* 2018). Indeed, *Kiss1* mRNA was reportedly more strongly expressed in rat GC than in TC and other ovarian cells (Ricu *et al.* 2012), an observation that concurs with our finding for small bovine follicles and with the finding of Basini *et al.* (2018) for kisspeptin immunoreactivity in preantral and antral porcine follicles. The apparent inconsistencies of the expression of kiss-1/Kiss1r in the ovary may relate to the variety of analytical methods used for detection and also to the use of ovarian tissues and cells from animals of different reproductive age, different oestrous/menstrual cycle stages and different species (Castellano *et al.* 2006, Gaytan *et al.* 2009, Shahed & Young 2009, Ricu *et al.* 2012, Mondal *et al.* 2015, Merhi *et al.* 2016, Basini *et al.* 2018).

It has been shown in rats (Laoharatchatathanin *et al.* 2015) and humans (Shahed & Young 2009, Mondal *et al.* 2015, 2016) that kisspeptin expression increased as follicles grow, with a peak level at the preovulatory stage. This increased expression most likely reflects the direct stimulatory effect of increased LH secretion from the anterior pituitary since, in rats, it can be blocked by treatment with a GnRH antagonist and restored by the administration of exogenous gonadotrophin (hCG) (Castellano *et al.* 2005). In the present study, there was no evidence for an increased expression of *KISS1* in LEA follicles, although, as mentioned above, relative expression levels of both *KISS1* and *KISS1R* mRNA were higher in TC than GC in large follicles, with this ratio reversed in small follicles.

Collectively, these findings raised the possibility that kisspeptin signalling may contribute to the regulation of follicular and/or luteal steroidogenesis. We therefore undertook a comprehensive series of dose–response experiments to examine the abilities of a kisspeptin agonist (kisspeptin-10) and antagonist (kisspeptin 234) to modulate steroid production and viable cell number in four different bovine follicular cell culture models (non-luteinized TC and GC; luteinized TC and GC). To our knowledge, there have been just a few studies examining possible direct effects of kisspeptin on ovarian steroidogenesis, and most of these involved culturing GC under luteinizing conditions. Kisspeptin treatment was shown to increase P4 production and expression of key steroidogenic enzymes (*Star, Cyp11a1* and* Hsd3b1*) by cultured rat luteal cells (Peng *et al.* 2013), while the kisspeptin antagonist (kisspeptin 234) reduced hCG-induced P4 secretion by cultured rat GC (Laoharatchatathanin *et al.* 2015). A kisspeptin-induced increase in P4 secretion and expression of *STAR, CYP11A1* and* HSD3B1* has also been reported in cultured chicken GC (Xiao *et al.* 2011). More recently, kisspeptin-10 was shown to increase P4 production and *STAR* mRNA expression by bovine GCs cultured in medium supplemented with 10% FCS (Guo *et al.* 2022). As such, these observations are indicative of a positive role for kisspeptin in promoting P4 synthesis, although possible effects on other ovarian steroids were not reported. With this in mind, the current study appears to be the first to examine *in vitro* whether kisspeptin-10 and a kisspeptin antagonist modulate basal and LH-induced secretion of A4 and P4 by non-luteinized TC, basal and FSH-induced secretion of E2 and P4 by non-luteinized GC and basal and FSK-induced secretion of P4 by luteinized TCs and GCs. Collectively, the results from this series of experiments were negative and, despite testing over a wide range of concentrations, neither kisspeptin-10 nor the kisspeptin antagonist (kisspeptin 234) elicited any significant change in the production of A4, P4 and E2 by the cells under basal or stimulated conditions. Furthermore, there was no effect of kisspeptin-10 or its antagonist on viable cell number at the end of culture, indicating a lack of effect on cell proliferation and/or survival. It should be noted that the amino acid sequence of bovine, rat, mouse and human kisspeptin-10 are identical, and so species differences in biopotency could not explain this lack of effect on bovine cells. In contrast to our negative findings for luteinized GC, kisspeptin-10 was recently reported to increase P4 production and *STAR* mRNA expression by bovine GCs cultured in serum-supplemented medium (Guo *et al.* 2022). However, cells were treated for a shorter time period (24 h) than the standard 96-h treatment period used in our model, and it is possible that this difference in culture duration and/or treatment exposure period influences the response. Further experiments involving a different experimental design would be needed to investigate this possibility.

Notwithstanding these negative findings, the possibility cannot be excluded that local kisspeptin signalling has other intraovarian roles. Indeed, several such roles have been suggested, based on experimental observation in other species (Hu *et al.* 2017, Cao *et al.* 2019). For instance, treatment of cultured mouse cumulus oocyte complexes with kisspeptin enhanced oocyte maturation (Chakravarthi *et al.* 2021), while mice with oocyte-specific deletion of *Kiss1r* showed premature ovulatory failure (Ruohonen *et al.* 2022). Moreover, intraovarian administration of kisspeptin antagonist to ageing rats led to a reduced number of CL, while intraovarian administration of kisspeptin increased CL number (Fernandois *et al.* 2016). The latter finding indicates a role for kisspeptin in the ovulatory process and is consistent with an earlier report that a marked fall in ovarian Kiss1 expression accompanied the disrupted ovulation in rats treated with inhibitors of prostaglandin synthesis (Gaytan *et al.* 2009).

In conclusion, the results show that *KISS1* and *KISS1R* mRNA are expressed in different bovine endocrine tissues, including pituitary, adrenal, testis and ovary (including follicular TC, GC and CL). Moreover, changing levels of expression were detected during different stages of follicle and corpus luteum development. Despite this evidence supporting the existence of an intraovarian kisspeptin–KISS1R system in the bovine ovary, an extensive series of *in vitro* experiments involving bovine ovarian cells in primary culture offered no evidence to support the hypothesis that kisspeptin–KISS1R signalling has a direct intraovarian role to modulate follicular or luteal steroidogenesis.

## Declaration of interest

The authors declare that there is no conflict of interest that could be perceived as prejudicing the impartiality of the research reported.

## Funding

DM was supported by a postgraduate scholarship from the Cultural Bureau of the Royal Embassy of Saudi Arabia. The work was supported, in part, by the BBSRC (grant no. BB/M001369 to PGK).

## Author contribution statement

PK and DM conceived the study, analysed the data and wrote the paper. DM performed experiments with contributions from MS and WC.
